# Characterization of Chloroplast Genomes From Two *Salvia* Medicinal Plants and Gene Transfer Among Their Mitochondrial and Chloroplast Genomes

**DOI:** 10.3389/fgene.2020.574962

**Published:** 2020-10-22

**Authors:** Chengwen Gao, Chuanhong Wu, Qian Zhang, Xia Zhao, Mingxuan Wu, Ruirui Chen, Yalin Zhao, Zhiqiang Li

**Affiliations:** Laboratory of Medical Biology, Medical Research Center, The Affiliated Hospital of Qingdao University, Qingdao, China

**Keywords:** *Salvia*, chloroplast genome, DNA barcodes, gene transfer, phylogenetic

## Abstract

*Salvia* species have been widely used as medicinal plants and have played an important role in the treatment and recovery of individuals with COVID-19. In this study, we reported two newly identified whole chloroplast genome sequences of *Salvia* medicinal plants (*Salvia yangii* and *Salvia miltiorrhiza* f. *alba*) and compared them with those of seven other reported *Salvia* chloroplast genomes. These were proven to be highly similar in terms of overall size, genome structure, gene content, and gene order. We identified 10 mutation hot spots (*trnK-rps16*, *atpH-atpI*, *psaA-ycf3*, *ndhC-trnV*, *ndhF*, *rpl32-trnL*, *ndhG-ndhI*, *rps15-ycf1*, *ycf1a*, and *ycf1b*) as candidate DNA barcodes for *Salvia*. Additionally, we observed the transfer of nine large-sized chloroplast genome fragments, with a total size of 49,895 bp (accounting for 32.97% of the chloroplast genome), into the mitochondrial genome as they shared >97% sequence similarity. Phylogenetic analyses of the whole chloroplast genome provided a high resolution of *Salvia*. This study will pave the way for the identification and breeding of *Salvia* medicinal plants and further phylogenetic evolutionary research on them as well.

## Introduction

*Salvia* L. species constitute the largest genus in the family Lamiaceae and comprise approximately 1,000 species worldwide. Of these, 84 *Salvia* species originated from southwestern China, especially from the Hengduan Mountains region (Li et al., 2013). The name *Salvia* comes from the Latin word “salvare,” which means “healing.” *Salvia* species have been widely used as medicinal plants, and as an important component of the Xuebijing injection, they have played an important role in the treatment and recovery of individuals with COVID-19 (Pan et al., 2020; Wen et al., 2020; Zhang Y.L. et al., 2020). For thousands of years in China, more than 40 *Salvia* species have been used for treating common cold, tuberculosis, bronchitis, hemorrhages, and menstrual disorders (Topcu, 2006). Among these, *Salvia miltiorrhiza* Bunge (Danshen), a characteristic traditional Chinese medicine (TCM) (Li et al., 2018), has been used for treating cerebrovascular and cardiovascular diseases effectively (Chen and Chen, 2017; Wang et al., 2017). However, compared with the purple-flowered *S. miltiorrhiza* variety, the white-flowered landrace of *Salvia miltiorrhiza* f. *alba* exerts superior medicinal qualities (Zhang et al., 2016). *Salvia yangii* also possesses high medicinal value (Mazandarani and Ghaemi, 2010). However, the difficulty in identifying these plants leads to an uneven quality of *Salvia* medicinal materials (Hu G.X. et al., 2016; Xiang et al., 2016; Drew et al., 2017). With the development of sequencing technology, the sequencing of chloroplast genomes can be fulfilled in a short time at a relatively low cost currently. Whole chloroplast genome sequences can provide more genetic information and higher species resolution ability than other molecular data, and they provide a possible resolution to these issues. However, the chloroplast genomes of most *Salvia* plants remain unknown.

Chloroplasts are specialized organelles where photosynthesis occurs and are composed of stacked thylakoids interconnected by lamellae (Douglas, 1998; Brunkard et al., 2015). The highly conserved chloroplast genomes possess a quadripartite structure including small single-copy (SSC) and large single-copy (LSC) regions along with dual copies of inverted repeat (IR) regions (Wicke et al., 2011; Wang et al., 2015). Whole chloroplast genomes provide crucial information, which can be used for understanding evolutionary differences, exploring genetic diversity, and generating highly resolved phylogenies, especially in complex/low taxonomic levels (Hu H. et al., 2016; Xu et al., 2019; Huang et al., 2020).

Here, we assembled the chloroplast genomes of *S. miltiorrhiza* f. *alba* and *S. yangii*, followed by comparing them with seven previously reported *Salvia* chloroplast genomes from GenBank. This study aimed to (1) perform a comparative analysis of the chloroplast genomes of these nine *Salvia* species; (2) ascertain the highly divergent sequences in the *Salvia* chloroplast genomes; (3) identify chloroplast gene insertion in the mitochondria; and (4) explore the evolutionary differences and similarities in the genus *Salvia* and lamiids. Thus, the information generated in this study would expand our knowledge on the evolution of plastome as well as the phylogenies of *Salvia* species.

## Materials and Methods

### Plant Material Sampling

The *Salvia* accessions in this study were from the *Salvia* nuclear genome sequencing project, and all plant materials were conserved in China Academy of Chinese Medical Sciences, Beijing, China (Xu et al., 2016; Boachon et al., 2018). The DNA was extracted from fresh leaves, followed by the use of Illumina sequencing technology to generate libraries. Post-sequencing, the paired-end sequence reads were obtained through next-generation sequencing.

### Chloroplast Genome Assembly and Annotation

Trimmomatic v0.38 was used to filter the raw sequencing data (Bolger et al., 2014). N-containing sequences and adapter sequences were removed. Sequences with a *Q* value less than 20 were also removed. Then, the clean data were used to perform *de novo* assembly using SPAdes 3.61 with varying *K*-mer parameters (Bankevich et al., 2012). We ordered *de novo* scaffolds that were positively correlated to the chloroplasts on the reference chloroplast genome of *S. miltiorrhiza* (NC_020431). Next, the Geneious Prime software v2020.0.4 (Kearse et al., 2012) was used to remap the paired-end reads to fill gaps in the final consensus sequence with multiple iterations.

GeSeq was used to conduct chloroplast genome annotation to predict transfer RNA (tRNA), gene-encoding proteins, and ribosomal RNA (rRNA), with manual adjustments as required (Tillich et al., 2017). We manually examined the IR junctions of *Salvia* species. A circular map of *Salvia* chloroplast genomes was subsequently drawn using OGDraw (Greiner et al., 2019).

### Genome Comparative Analysis and Identification of Hypervariable Regions

MAFFT 7.221 was used to align the chloroplast genome sequences of *S. miltiorrhiza* f. *alba* and *S. yangii* (Katoh and Standley, 2013). Next, DnaSP 6.12 was used to perform a sliding window analysis with a step size of 200 bp and window length of 600 bp in order to detect the rapidly evolving molecular markers for performing phylogenetic analysis (Librado and Rozas, 2009).

Tree-based methods were utilized to assess the hypervariable barcodes and to compare the chloroplast genes *matK* and *rbcL*. MEGA 7.0 software was used to build maximum likelihood (ML) trees with 1,000 bootstrap replicates for each hypervariable marker (Kumar et al., 2016).

### Identification of Chloroplast Gene Insertion in the Mitochondria

The mitochondrial genome of *S. miltiorrhiza* was retrieved from GenBank (NC_023209), followed by a homology search between chloroplast and mitochondria genomes using BLAST (with default parameters) to identify the transferred genes between the mitochondrial and chloroplast genomes. Circos was used to draw the chloroplast and mitochondrial maps from *Salvia* species, along with the fragments of gene transfers (Krzywinski et al., 2009).

### Phylogenetic Analysis

We used the following three methods to perform phylogenetic analyses of *Salvia* species: Bayesian inference (BI) with a GTR + I + G model using MrBayes 3.2 [the Markov chain Monte Carlo (MCMC) algorithm was run for 1 million generations and sampled every 100 generations] (Ronquist et al., 2012); maximum likelihood (ML) using MEGA 7.0 with 1,000 bootstrap replicates (Kumar et al., 2016); and maximum parsimony (MP) with a heuristic search in PAUP 4.0 with 1,000 random taxon stepwise addition sequences (Swofford, 1993).

## Results

### Chloroplast Genome Organization and Features

**FIGURE 1:**
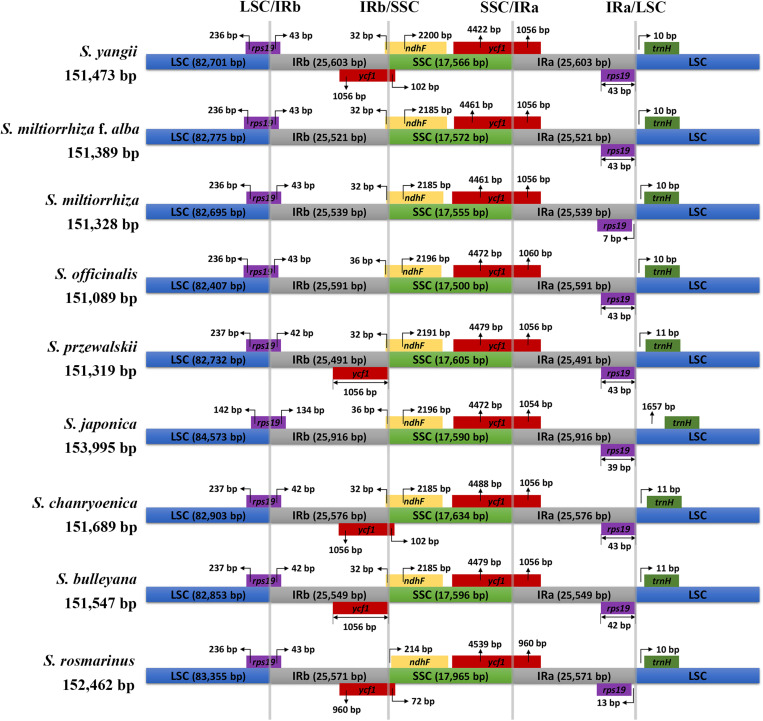
Comparison of inverted repeat (IR)/small single-copy (SSC) expansions in *Salvia* species. *Colored boxes above the horizontal line* indicate the genes and gene segments across the IRa/b junctions. IR segments and genes are not to scale.

The chloroplast genomes of *S. yangii* and *S. miltiorrhiza* f. *alba* were 151,473 and 151,389 bp long, respectively, and exhibited a quadripartite structure with dual IR regions (25,603 and 25,521 bp), an SSC region (17,566 and 17,572 bp), and an LSC region (82,701 and 82,775 bp) (Figure 1). *S. yangii* and *S. miltiorrhiza* f. *alba* contained 116 and 115 unique genes in their chloroplast genomes, respectively, of which, 82 and 81 were protein-coding genes, four were rRNAs, and 30 were tRNAs (Table 1). Eighteen genes exhibited introns, of which six tRNAs (*trnV-UAC*, *trnG-UCC*, *trnK-UUU*, *trnL-UAA*, *trnI-GAU*, and *trnA-UGC*) and nine protein-coding genes (*accD*, *rps16*, *rpl16*, *ndhB*, *petB*, *rpl2*, *atpF*, *rpoC1*, and *ndhA*) had a single intron and the remaining three genes displayed two introns (*ycf3*, *rps12*, and *clpP*). For both *S. yangii* and *S. miltiorrhiza* f. *alba*, the *trnK-UUU* intron, comprising the *matK* gene, had the largest intron for both species (2,515 and 2,522 bp, respectively). Furthermore, the *Salvia* chloroplast genomes possessed similar GC contents (38.0–38.1%). IR expansion was considered the main cause for the size differences in the *Salvia* chloroplast genomes. Both *Salvia* species were found to be generally conserved in terms of gene order and genome structure (Table 1). The GenBank accession numbers for the complete chloroplast genome sequences of the two *Salvia* species were MT012420 and MT012421.

**TABLE 1:** Summary of the features of the chloroplast genome of nine *Salvia* species.

Species	Genome size (bp)	LSC length (bp)	IR length (bp)	SSC length (bp)	GenBank number	GC%	Number of genes			
							Total	CDS	rRNA	tRNA
*S. yangii*	151,473	82,701	25,603	17,566	MT012421	38.1	116	82	4	30
*S. miltiorrhiza* f. *alba*	151,389	82,775	25,521	17,572	MT012420	38.0	115	81	4	30
*S. miltiorrhiza*	151,328	82,695	25,539	17,555	NC_020431	38.0	115	81	4	30
*S. officinalis*	151,089	82,407	25,591	17,500	NC_038165	38.0	115	81	4	30
*S. przewalskii*	151,319	82,732	25,491	17,605	NC_041091	38.0	115	81	4	30
*S. japonica*	153,995	84,573	25,916	17,590	NC_035233	38.0	115	81	4	30
*S. chanryoenica*	151,689	82,903	25,576	17,634	NC_040121	38.0	115	81	4	30
*S. bulleyana*	151,547	82,853	25,549	17,596	NC_041092	38.0	115	81	4	30
*S. rosmarinus*	152,462	83,355	25,571	17,965	NC_027259	38.0	115	81	4	30

Comparisons of the fully annotated IR/SC junction regions were found to exhibit almost the same relative positions among the nine *Salvia* chloroplast genomes (Figure 1). The *rps19* gene contained all LSC/IRb junctions, resulting in the partial expansion (42–134 bp) of the IRb region toward the *rps19* gene. The IRb/SSC boundary positions were located on the *ycf1* and *ndhF* genes, and the SSC/IRa borders in the nine chloroplast genomes were located on the *ycf1* gene.

### Comparative *Salvia* Chloroplast Genomes and Divergence Hot Spot Regions

**FIGURE 2:**
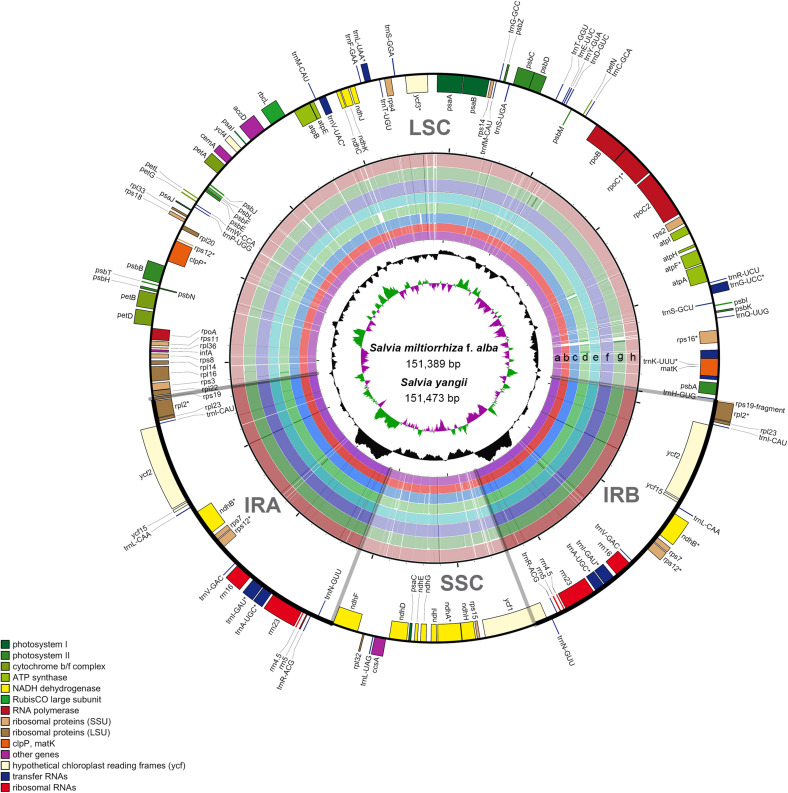
Map of the aligned *Salvia* chloroplast genomes. Gene map of the *Salvia* chloroplast genomes, sequence alignment of *Salvia* species chloroplast genomes [*S. miltiorrhiza* f. *alba* (a); *S. yangii* (b); *S. officinalis* (c); *S. japonica* (d); *S. chanryoenica* (e); *S. bulleyana* (f); *S. rosmarinus* (g); *S. przewalskii* (h), with *S. miltiorrhiza* as the reference], GC content, and GC skew from outside to inside (the genes that are transcribed clockwise are depicted *on the outside of the circle*, while those transcribed counterclockwise are depicted *inside*). *Indicate genes exhibited introns.

The results of the comprehensive sequence divergence of the two newly assembled and the seven previously reported *Salvia* chloroplast genomes with *S. miltiorrhiza* as the control displayed high sequence similarity (Figure 2). As expected, the LSC and SSC regions exhibited comparatively higher sequence divergence than did the IR regions. A search for nucleotide substitutions identified 5,833 variable sites (3.69%), including 2,486 parsimony-informative sites (1.57%), across the nine *Salvia* chloroplast genomes.

**FIGURE 3:**
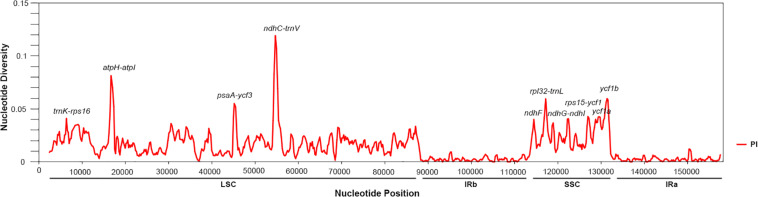
Sliding window analysis of the *Salvia* chloroplast genomes (step size, 200 bp; window length, 600 bp). *X*-axis: midpoint position of the window; *Y*-axis: nucleotide diversity in each window.

Next, in the *Salvia* chloroplast genomes, we calculated the nucleotide diversity (*π*) values within 600-bp windows to detect the sequence divergence hot spots. The *π* values were in the range of 0–0.119, with extremely high values (*π* > 0.002) in the following 10 regions (*trnK-rps16*, *atpH-atpI*, *psaA-ycf3*, *ndhC-trnV*, *ndhF*, *rpl32-trnL*, *ndhG-ndhI*, *rps15-ycf1*, *ycf1a*, and *ycf1b*) (Figure 3 and Table 2). Divergence hot spot regions could be the ideal molecular markers to distinguish *Salvia* species. Two conventional candidate DNA barcodes (*matK* and *rbcL*) were used to compare the marker divergence. The results revealed a lower variability in these barcodes compared with the newly identified markers (Table 2). The *ndhC-trnV* region exhibited the highest variability (38.75%). Supplementary Figure S1 presents the graphical representation of these results using the ML method.

**TABLE 2:** Variability in the two universal chloroplast DNA barcodes and the 10 novel markers in *Salvia* species.

Markers	Length (bp)	Variable sites		PI sites^a^		Nucleotide diversity	Number of haplotypes
		Number	%	Number	%		
*trnK-rps16*	557	60	10.77	31	5.57	0.041	9
*atpH-atpI*	840	203	24.17	36	4.29	0.067	8
*psaA-ycf3*	625	117	18.72	18	2.88	0.052	8
*ndhC-trnV*	862	334	38.75	31	3.60	0.096	9
*ndhF*	2,217	129	5.82	68	3.07	0.022	8
*rpl32-trnL*	619	97	15.67	47	7.59	0.058	9
*ndhG-ndhI*	339	53	15.63	31	9.14	0.061	9
*rps15-ycf1*	369	51	13.82	29	7.86	0.053	8
*ycf1a*	974	58	5.95	27	2.77	0.020	9
*ycf1b*	1,024	115	11.23	47	4.59	0.039	8
*rbcL*	1,446	37	2.56	19	1.31	0.009	7
*matK*	1,535	90	5.86	49	3.19	0.023	9

*^a^Parsimony informative sites.*

### Characterization of *Salvia* Chloroplast Genome Transfer Into the Mitochondrial Genome

**FIGURE 4:**
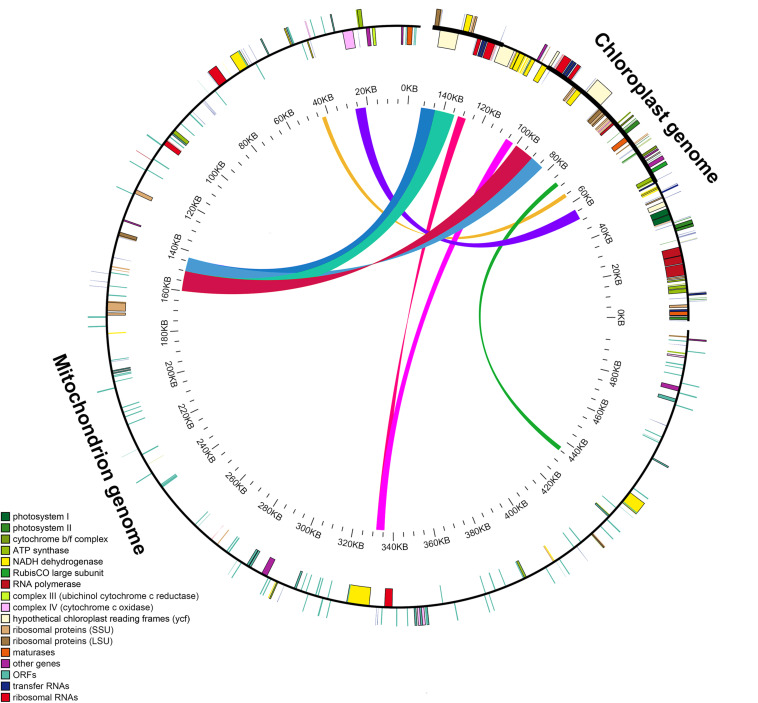
Schematic for the chloroplast-to-mitochondrial gene transfer in *Salvia* species. A *colored line within the circle* represents the areas of the chloroplast genome that were transferred across the specified position in the mitochondrial genome. Genes shown *inside* and *outside the circle* have been transcribed in a clockwise and counterclockwise manner, respectively.

The length of the GenBank mitochondrial genome sequence of *S. miltiorrhiza* (499,236 bp) was found to be approximately 3.3 times longer than that of the chloroplast genome. We identified nine large chloroplast genome fragments in the mitochondrial genome, including both genes and intergenic regions. The fragments ranged from 1,737 to 9,734 bp and retained >97% of their sequence identity with their original chloroplast counterparts. These fragments had a total length of 49,895 bp, accounting for ∼32.97% of the chloroplast genome (Figure 4 and Supplementary Table S1). Eleven intact chloroplast genes (*ndhB*, *rps7*, *rps12*, *ycf2*, *rpl23*, *atpE*, *atpB*, *rbcL*, *psbB*, *petL*, and *petG*), six tRNAs (*trnL-CAA*, *trnV-GAC*, *tRNA-Ile*, *trnM-CAU*, *trnW-CCA*, and *trnP-UGG*), one rRNA (*rrn23*), and numerous partial genes and intergenic spacer regions were identified.

### Phylogenetic Analysis

**FIGURE 5:**
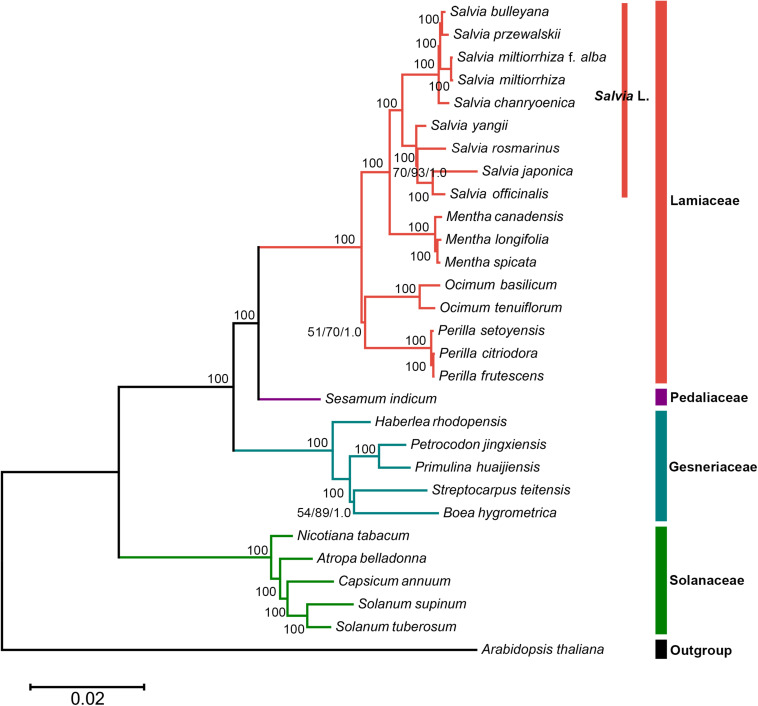
The whole chloroplast genome sequence-based maximum likelihood (ML) phylogenetic tree of the nine *Salvia* species with 19 related species in the lamiids. *Numbers posted with branches* are the ML bootstrap values, maximum parsimony (MP) bootstrap values, and Bayesian posterior probabilities, respectively. *100* indicate 100% ML and MP bootstrap support and 1.0 Bayesian posterior probability.

In this study, the phylogenetic position of *Salvia* in the lamiids was standardized using chloroplast genomes (Figure 5). Three separate methods were used for conducting the phylogenetic analyses of the chloroplast genomes: ML, BI, and MP. Approximately identical topologies were generated by the ML and BI methods, with a high support for the majority of the branches (Supplementary Figure S2). However, marginally different positions of a few taxa were revealed by the MP trees compared with those by ML and BI (Supplementary Figure S3). Despite these variations, a majority of taxonomical relationships were highly supported and well resolved, which supported that the use of chloroplast genomic data promoted the resolution of phylogenetic analyses. Thus, the phylogenetic tree showed that *S. miltiorrhiza* f. *alba*, *S. miltiorrhiza*, *Salvia bulleyana*, and *Salvia przewalskii* were clustered on a single terminal branch.

## Discussion

### Characterization of Complete Chloroplast Genome Structures

This is the first study to report the complete chloroplast genome of *S. yangii* and *S. miltiorrhiza* f. *alba*. We found the genomic structures along with gene type, number, and order to be fairly identical among the nine *Salvia* chloroplast genomes, with the exception of variations in the numbers of SNPs and small Indels (Qian et al., 2013; He et al., 2017; Du et al., 2019; Liang et al., 2019; Tao et al., 2019; Zhang X.J. et al., 2020). Thus, the study of the complete chloroplast genomes would afford useful genetic information to study the authentication, breeding, and evolutionary similarities/differences between *Salvia* species.

### Comparative Analysis of *Salvia* Chloroplast Genomes and DNA Barcodes

The chloroplast genomes were found to be fairly conserved across the nine taxa, except for a few variable regions, based on the results of the comparative analysis. The coding regions contained most of the conserved sequences, whereas the non-coding regions had most of the variable sequences. Consistent with the results of similar studies on other plants, the LSC and SSC regions were less conserved than the IR region (Figure 1; Song et al., 2019).

DNA barcoding was first proposed in 2003 (Hebert et al., 2003). DNA barcoding is a new technique that is widely used as a biological tool for breeding, species identification, and evolutionary research (Techen et al., 2014; Mishra et al., 2016). The herbal medicine industry relies on the identification of novel plant species. Due to their high medicinal value and cost-effective processing, it has become necessary to develop easy and safe methods for the identification and development of *Salvia* species.

The chloroplast genomes possess a smaller size and multiple copies in a cell compared with the nuclear genome. Furthermore, due to the presence of adequate interspecific divergence, in chloroplast genomes, the best species authentication methods are based on chloroplast genome-based DNA barcodes (Hollingsworth et al., 2011).

Here, we found an increase in the number of variable sites in the following 10 specific regions based on the results of pairwise chloroplast genomic alignment and SNP analysis: *trnK-rps16*, *atpH-atpI*, *psaA-ycf3*, *ndhC-trnV*, *ndhF*, *rpl32-trnL*, *ndhG-ndhI*, *rps15-ycf1*, *ycf1a*, and *ycf1b*. Thus, *Salvia* species may be detected using these regions as novel candidate fragments. However, further experiments are required to support this *Salvia* chloroplast sequence data.

### Chloroplast Genome Fragments Were Found in the Mitochondrial Genome

Specific information pertaining to the intracellular gene transfer between different genomes (mitochondrial, nuclear, and chloroplast) has been disclosed through sequencing analysis (Timmis et al., 2004; Nguyen et al., 2020). Previous research has detected high amounts of transfer of nuclear DNA from the organelle in angiosperms (Hazkani-Covo et al., 2010; Smith, 2011; Park et al., 2014). Additionally, a characteristic feature of long-term evolution has been identified as chloroplast-to-mitochondrial gene transfer (Gui et al., 2016; Nguyen et al., 2020). Here, in *Salvia* species, we identified nine large chloroplast genome (32.97% of the chloroplast genome) fragments in the mitochondrial genomes.

### Phylogenetic Analysis of *Salvia* Species

The whole chloroplast genome sequence-based phylogenetic tree was built to explore the evolutionary similarities/differences between *Salvia* species and between genera in the lamiids. We found that *S. przewalskii*, *S. miltiorrhiza*, *S. bulleyana*, and *S. miltiorrhiza* f. *alba* were clustered on a single terminal branch. Several studies have revealed similarities in appearance and characteristics between these species (Li et al., 2008; Wang et al., 2014). Regarding their compositions, common substituents of *S. miltiorrhiza* (*S. bulleyana*, *S. przewalskii*, and *S. miltiorrhiza* f. *alba*) shared the maximum chemical composition of *S. miltiorrhiza* (Danshen) (Wang et al., 2018).

## Data Availability Statement

The data that support the findings of this study are openly available in GenBank of NCBI at https://www.ncbi.nlm.nih.gov, reference number MT012420 and MT012421. Chloroplast genome raw sequencing data have been deposited at the NCBI Sequence Read Archive (SRA) under accession PRJNA646330.

## Author Contributions

ZL and CG conceived the study and acquired the funding. CG performed the data analyses and drafted the earlier version of the manuscript. CW, QZ, XZ, MW, RC, and YZ revised the manuscript. All authors read and approved the final manuscript.

## Conflict of Interest

The authors declare that the research was conducted in the absence of any commercial or financial relationships that could be construed as a potential conflict of interest.
