# Supplementary figures and images for: Characterization of Chloroplast Genomes From Two Salvia Medicinal Plants and Gene Transfer Among Their Mitochondrial and Chloroplast Genomes
Source: Front Genet. 2020 Oct 22;11:574962. doi: 10.3389/fgene.2020.574962 (PMC7642825; doi:10.3389/fgene.2020.574962)

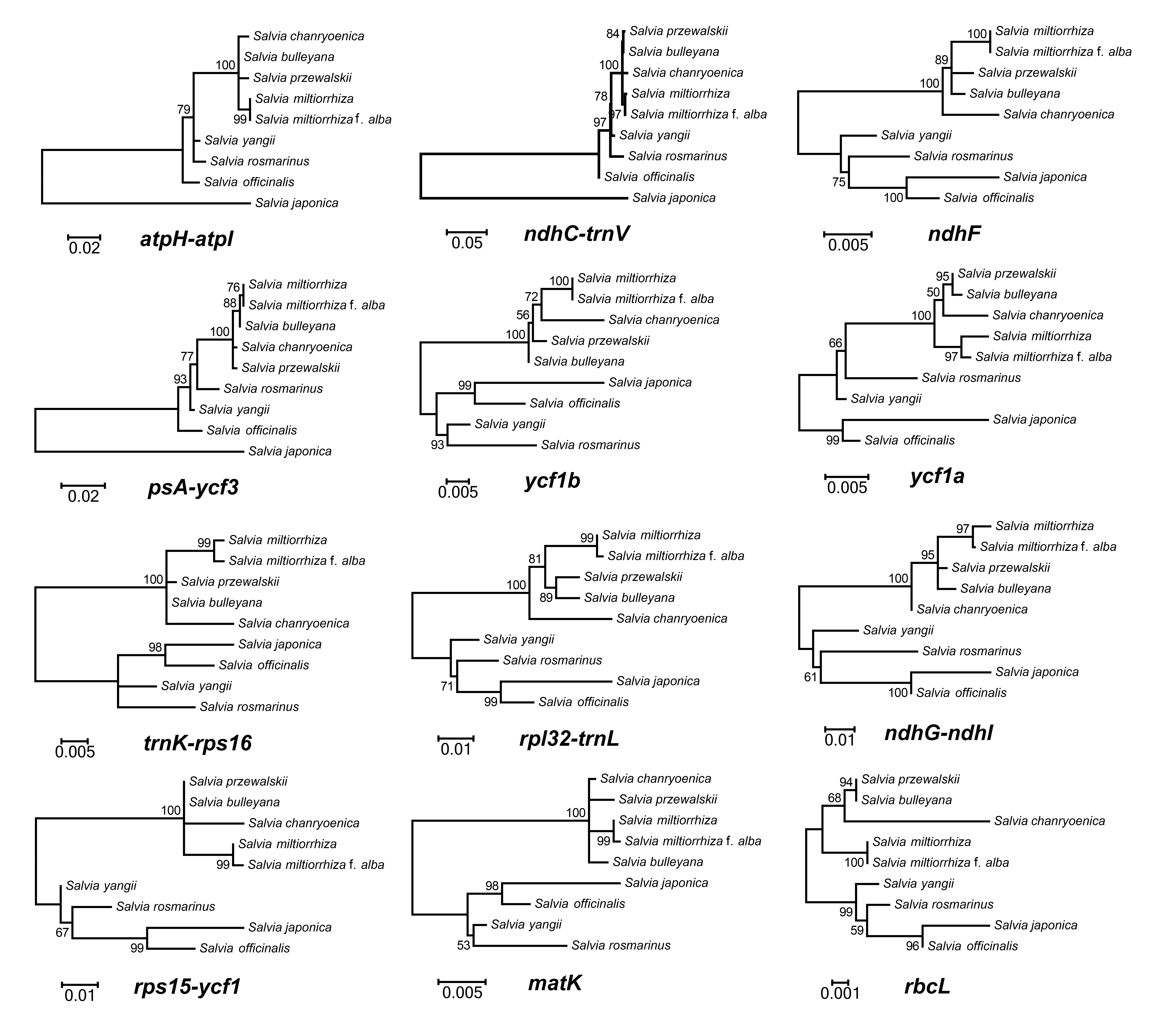

Supplement: Supplementary file 1 [file Image_1.TIF]

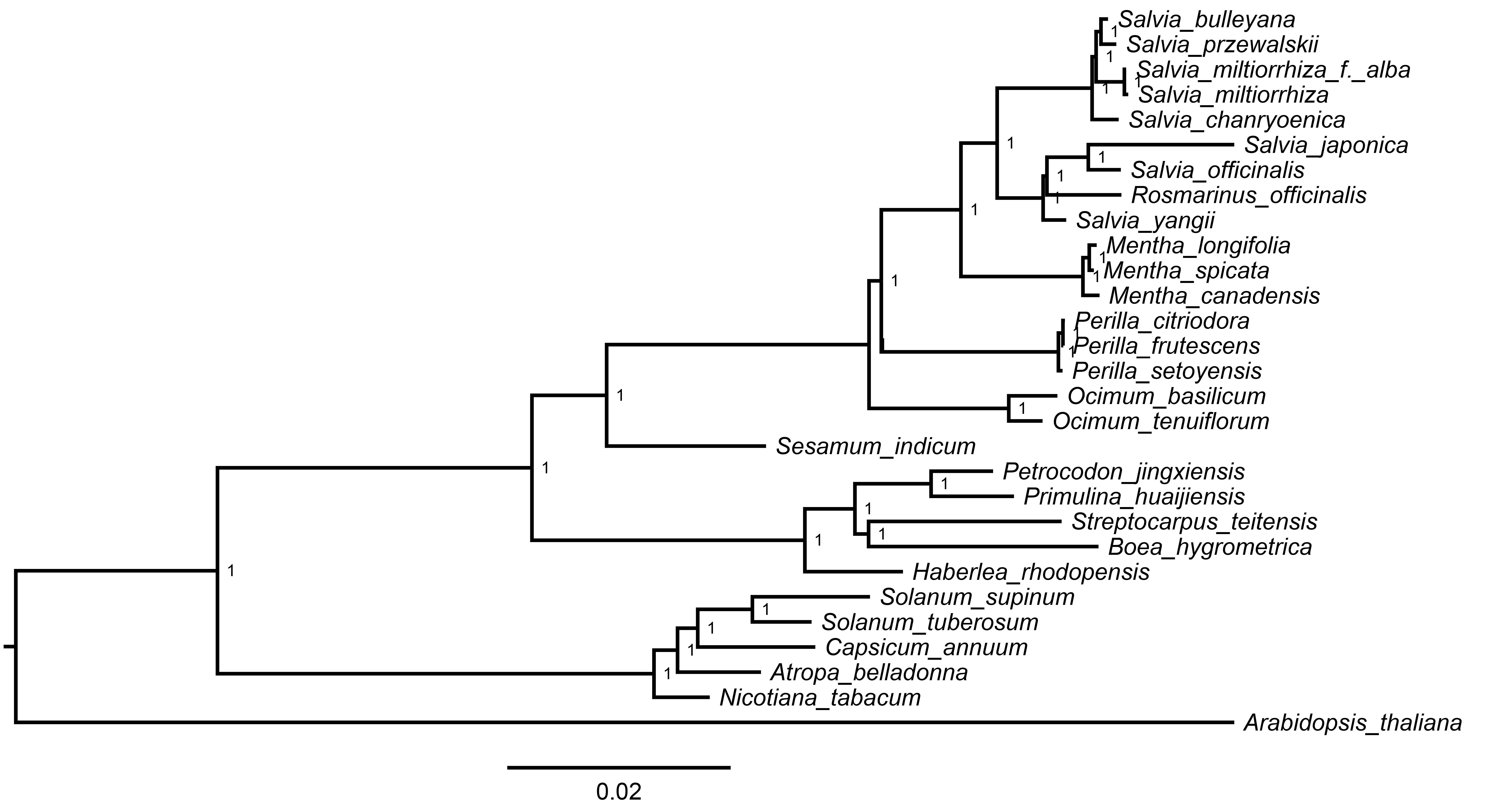

Supplement: Supplementary file 2 [file Image_2.TIF]

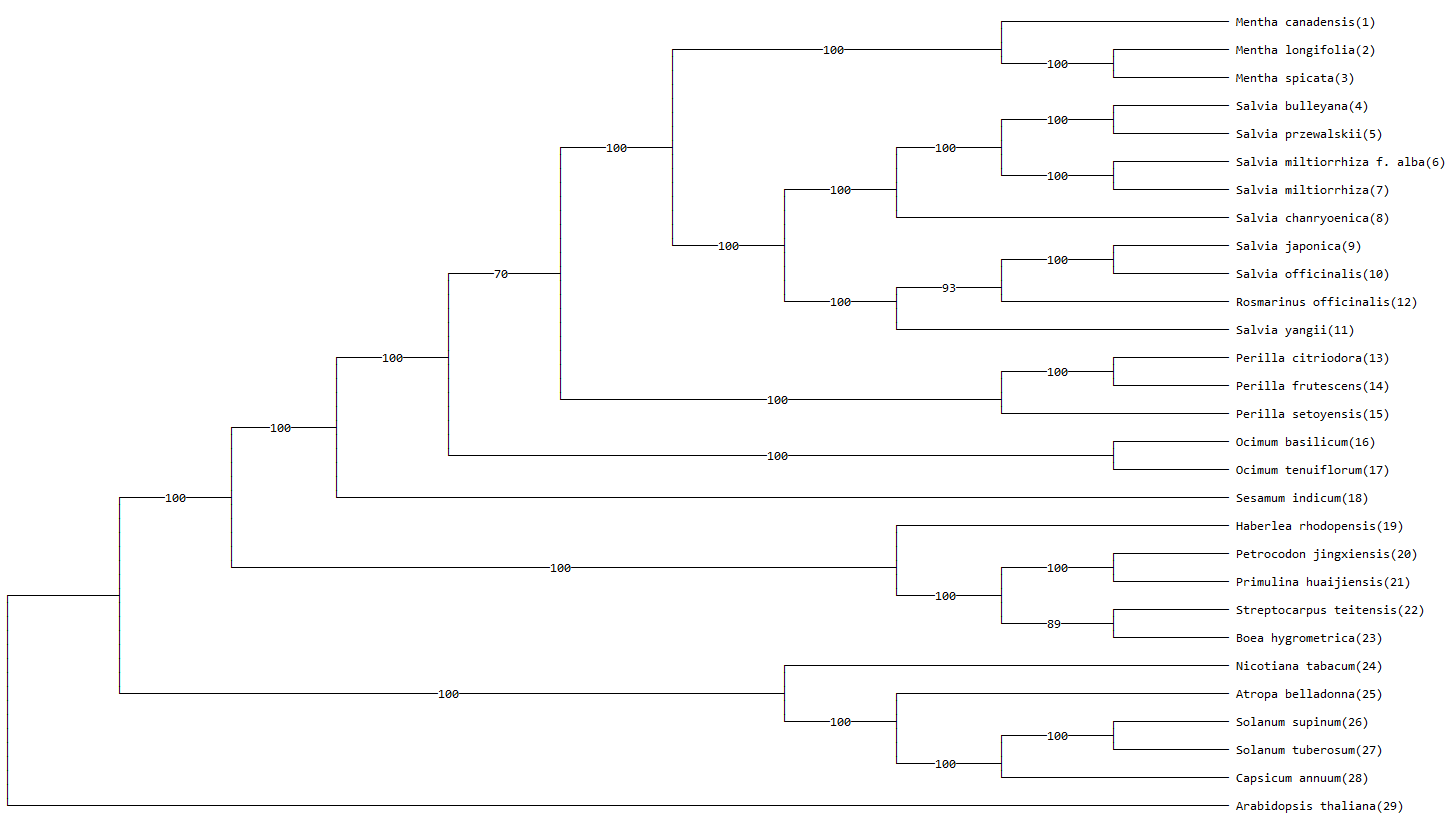

Supplement: Supplementary file 3 [file Image_3.TIF]
