# Supplementary material for: Characterization of Chloroplast Genomes From Two Salvia Medicinal Plants and Gene Transfer Among Their Mitochondrial and Chloroplast Genomes
Source: Front Genet. 2020 Oct 22;11:574962. doi: 10.3389/fgene.2020.574962 (PMC7642825; doi:10.3389/fgene.2020.574962)
Supplement: Supplementary file 5 [file Table_2.DOCX]

Supplementary Material

## Supplementary Figures

**Supplementary Figure 1.** Phylogenetic tree of *Salvia* species using maximum likelihood (ML) methods based on ten novel markers and the two universal plastid DNA barcodes.

**Supplementary Figure 2.** Phylogenetic trees of lamiids based on Bayesian analysis.

**Supplementary Figure 3.** Phylogenetic trees of lamiids based on maximum parsimony (MP) analysis.

**Supplementary table 1.** Alignment results between Salvia mitochondrial and chloroplast genomes.
